# Biological Effects of Umbelliferone in MCF-7 Breast Cancer Cells: Integrated In Vitro and Computational Analyses

**DOI:** 10.3390/ph19101536

**Published:** 2026-09-28

**Authors:** Şerife Gökçe Çalışkan

**Affiliations:** Department of Physics, Faculty of Sciences, Aydın Adnan Menderes University, 09010 Aydın, Türkiye; gcaliskan@adu.edu.tr; Tel.: +90-532-322-53-28

**Keywords:** umbelliferone, MCF-7, breast cancer, apoptosis, caspase activity, molecular docking, molecular dynamics, ADME

## Abstract

**Background/Objectives:** Breast cancer remains a leading cause of cancer-related mortality among women, highlighting the need for further investigation of bioactive compounds with selective effects on cancer cells. This study evaluated the biological effects of the natural coumarin derivative umbelliferone in MCF-7 breast cancer cells using integrated in silico and in vitro approaches. **Methods:** Molecular docking predicted interactions with AKT1, mTOR, PI3Kα, and Caspase-7, while molecular dynamics simulations and MM/PBSA calculations provided further information on interactions with AKT1 and mTOR. **Results:** Umbelliferone reduced MCF-7 cell viability in a concentration-dependent manner, with an IC_50_ of 492 µM after 24 h, whereas 50% viability was not reached in BJ normal fibroblasts up to 1400 µM, corresponding to an IC_50_ > 1400 µM and a selectivity index > 2.85. Annexin V-FITC/PI analysis showed increased apoptotic populations, with total apoptosis significantly increased at 800 µM. Colony formation analysis further indicated reduced clonogenic capacity. At 400 µM, umbelliferone significantly increased relative Caspase-3/7 and Caspase-9 activities to 1.873 ± 0.635 and 2.483 ± 0.092, respectively, whereas neither activity was significantly increased at 800 µM. In silico ADME analysis predicted properties compatible with commonly used drug-likeness criteria. **Conclusions:** Collectively, the findings demonstrate effects of umbelliferone on cell viability, apoptosis, clonogenic capacity, and caspase activity in MCF-7 cells, with comparatively lower cytotoxicity toward normal fibroblasts. Computational findings remain hypothesis-generating and do not establish direct target engagement or pathway inhibition. Further mechanistic and in vivo studies are required to determine the biological and pharmacological relevance of these findings.

## 1. Introduction

Breast cancer is among the most frequently diagnosed malignancies in women and continues to represent a major global health challenge, largely due to tumor heterogeneity and the emergence of treatment resistance [1]. Accumulating evidence indicates that dysregulation of intracellular signaling pathways plays a central role in tumor cell proliferation, survival, metastasis, and resistance to therapy. Among these pathways, the PI3Kα/AKT/mTOR signaling pathway has attracted particular attention, as it regulates critical processes involved in tumor development and treatment response, including cell differentiation, proliferation, energy metabolism, angiogenesis, cell-cycle progression, and inhibition of apoptosis [2]. Notably, aberrant activation of this pathway has been reported at high frequency in several cancers, including breast cancer [3]. In breast tumors, persistent PI3Kα/AKT/mTOR signaling is strongly associated with therapeutic resistance, contributing to reduced responsiveness to hormone therapy in hormone receptor-positive (HR^+^) disease and to chemotherapy resistance in both HR^+^ and triple-negative breast cancer (TNBC) subtypes [4,5,6].

The PI3Kα/AKT/mTOR signaling cascade is hierarchically organized, with phosphoinositide 3-kinase (PI3K) functioning upstream to activate AKT, a central survival kinase that subsequently regulates downstream effectors such as mTOR [7,8]. Dysregulation of this pathway represents one of the most frequent oncogenic alterations in breast cancer and has been closely associated with tumor progression and aggressive behavior. Inhibition of PI3Kα/AKT signaling is mechanistically linked to activation of the intrinsic apoptotic pathway. Reduced AKT activity may relieve the suppression of pro-apoptotic signaling, thereby promoting activation of the initiator Caspase-9 and subsequent activation of downstream executioner caspases [9,10]. Notably, MCF-7 cells are deficient in Caspase-3 expression; therefore, Caspase-7 represents a particularly relevant executioner caspase in this cellular model [11,12]. Accordingly, evaluating the potential interaction of umbelliferone with Caspase-7, together with PI3Kα, AKT1, and mTOR, provides a more biologically relevant framework for investigating its potential interactions with proteins associated with cell survival and apoptotic signaling. Among the class I PI3K isoforms, PI3Kα was selected in the present study because it represents the most frequently altered and clinically relevant isoform in breast cancer, particularly in HR^+^ subtypes [2]. Therefore, PI3Kα was considered a representative upstream component of the signaling cascade for computational evaluation.

The clinical relevance of targeting the PI3K/AKT/mTOR signaling axis in breast cancer has been increasingly demonstrated by the development of pathway-directed therapies. The PI3Kα-selective inhibitor alpelisib has established PI3Kα as a clinically actionable target in *PIK3CA*-mutated HR^+^/HER2^−^ advanced breast cancer, while the more recent introduction of the AKT inhibitor capivasertib in combination with fulvestrant has further expanded therapeutic targeting of this signaling network in patients with *PIK3CA/AKT1/PTEN*-altered HR^+^/HER2^−^ advanced disease [13,14]. These clinical advances underscore the therapeutic relevance of the PI3K/AKT signaling axis while also highlighting the importance of continued investigation of compounds capable of modulating cancer-associated signaling networks. In this context, natural products represent a chemically diverse source of bioactive scaffolds; however, their translation from preclinical activity to clinically useful anticancer agents remains challenging [15,16]. Limitations including poor aqueous solubility, limited bioavailability, rapid metabolism, variable target selectivity, and the frequent discrepancy between effective in vitro concentrations and achievable systemic exposure may restrict their therapeutic applicability [16]. Consequently, promising in vitro activity of natural compounds should be interpreted cautiously and supported by pharmacokinetic, mechanistic, in vivo, and ultimately clinical validation.

Despite these translational challenges, natural products and phytochemicals continue to attract considerable interest as potential sources of anticancer compounds, an approach that is also compatible with the principles of green chemistry. Green chemistry promotes the development of sustainable and environmentally conscious chemical processes and encourages the exploration of naturally derived molecular scaffolds [17]. Natural products offer substantial structural diversity and a broad spectrum of biological activities, and many natural metabolites exhibit multi-target effects that may be relevant to the regulation of complex signaling networks involved in cancer progression.

Umbelliferone (7-hydroxycoumarin) is a naturally occurring coumarin derivative widely distributed in medicinal plants and has been extensively reported to possess antioxidant [18], anti-inflammatory [19], anti-viral [20], and anti-tumor properties [21]. Increasing in vitro evidence demonstrates cytotoxic effects of umbelliferone against various cancer cell lines, including MCF-7 breast cancer cells [22]. Proposed mechanisms include activation of mitochondrial apoptotic pathways, inhibition of cancer cell migration, and modulation of the tumor microenvironment [23].

Given the continuing need for anticancer agents with improved efficacy and selectivity, the present study aimed to investigate the biological activity of umbelliferone in MCF-7 breast cancer cells through an integrated in silico and in vitro approach. Cytotoxic activity and selectivity were evaluated using the MTT assay in MCF-7 breast cancer cells and BJ normal fibroblasts, respectively, while apoptosis induction was assessed using Annexin V-FITC/PI flow cytometry. Caspase activity assays were additionally performed to further characterize apoptosis-associated signaling. The long-term antiproliferative effect of umbelliferone was further investigated using a colony formation assay. In parallel, molecular docking analyses were performed to investigate the potential interactions of umbelliferone with AKT1, mTOR, PI3Kα, and Caspase-7, while molecular dynamics simulations and MM/PBSA calculations were used to further characterize selected protein–ligand complexes. In silico ADME analysis was additionally conducted to predict the physicochemical, drug-likeness, and pharmacokinetic properties of umbelliferone. By integrating complementary experimental and computational approaches, this study provides a broader assessment of the biological activity of umbelliferone in MCF-7 cells and explores potential molecular interactions that may provide a basis for future mechanistic and pharmacological investigations.

## 2. Results

### 2.1. Molecular Docking and Interactions

Molecular docking analyses indicated that umbelliferone exhibited predicted interactions with AKT1, mTOR, PI3Kα, and Caspase-7, with calculated docking scores of −7.32, −6.98, −6.23, and −6.91 kcal/mol, respectively. To provide a methodologically consistent comparative context, the corresponding co-crystallized reference ligands were docked using the same AutoDock Vina (version 1.2.7) protocol applied to umbelliferone. OR4, ADP, X6K, and Ac-DNLD-CHO exhibited predicted docking scores of −10.71, −7.14, −9.82, and −6.70 kcal/mol toward AKT1, mTOR, PI3Kα, and Caspase-7, respectively. The co-crystallized ligands showed more favorable predicted docking scores than umbelliferone for AKT1, mTOR, and PI3Kα, whereas umbelliferone yielded a slightly more favorable predicted score than Ac-DNLD-CHO for Caspase-7.

Umbelliferone was further compared with established target-directed inhibitors under identical AutoDock Vina conditions. Capivasertib, AZD8055, alpelisib, and Ac-DNLD-CHO exhibited predicted docking scores of −8.43, −9.21, −9.24, and −6.70 kcal/mol toward AKT1, mTOR, PI3Kα, and Caspase-7, respectively. The established inhibitors showed more favorable predicted docking scores than umbelliferone for AKT1, mTOR, and PI3Kα. In contrast, the predicted docking score of umbelliferone toward Caspase-7 (−6.91 kcal/mol) was slightly more favorable than that of the co-crystallized reference inhibitor Ac-DNLD-CHO (−6.70 kcal/mol). However, given the small magnitude of this difference and the inherent limitations of docking-based scoring functions, this observation should not be interpreted as evidence of greater binding affinity or inhibitory potency.

Cisplatin was included as a positive control in the in vitro cytotoxicity experiments. However, because its primary anticancer mechanism involves DNA crosslinking rather than direct inhibition of the investigated kinase targets, docking-score comparison between cisplatin and umbelliferone was not considered an appropriate mechanistic benchmark for PI3Kα/AKT/mTOR-associated proteins. Accordingly, the computational findings were evaluated using the co-crystallized reference ligands and established target-directed inhibitors described above.

Analysis of the predicted protein–ligand interactions showed that umbelliferone formed conventional hydrogen bonds with SER205, THR211, and ASN204 and hydrophobic interactions with LEU210, TRP80, and LEU264 in AKT1. A π–π stacking interaction with TRP80 was also observed. In the mTOR complex, hydrogen bonds were detected with ASN1898, ASN1899, LEU1900, and GLN1937, together with π–σ and π–alkyl interactions involving LEU1900 and LEU2204. For PI3Kα, umbelliferone formed a hydrogen bond with THR462, together with π–σ and π–cation interactions involving ARG401. In the Caspase-7 complex, hydrogen bonding with THR463, interactions of π–anion with GLU516, π–sulfur with MET594 and π–alkyl with PRO227 were observed (Table 1, Figure 1).

Several residues involved in the predicted interactions of umbelliferone overlapped with those interacting with the corresponding co-crystallized ligands for AKT1 and mTOR, supporting the plausibility of the predicted binding orientations (Table 2). In contrast, no direct residue overlaps were observed for PI3Kα and Caspase-7, indicating a distinct predicted interaction pattern within the investigated binding region.

To independently validate the docking protocol, the selected co-crystallized ligands were re-docked into their respective crystallographic binding sites using AutoDock 4.2. The resulting RMSD values for AKT1, mTOR, PI3Kα, and Caspase-7 were 1.37, 1.52, 1.44, and 1.15 Å, respectively. All values were below the commonly accepted threshold of 2 Å, indicating satisfactory reproduction of the crystallographic binding poses and supporting the reliability of the docking protocol [24].

Overlap was defined as identical residues interacting with both umbelliferone and the corresponding co-crystallized ligand under the same interaction criteria. Lack of overlap does not preclude binding within the same active region, as ligands may occupy alternative microenvironments of the pocket.

### 2.2. Molecular Dynamics Results

During molecular docking, the ligand is typically treated as flexible while the target protein is considered rigid. However, under physiological conditions, both molecules exhibit dynamic behavior. In addition, many important interactions that occur in a fluctuating biological environment may be overlooked in static docking approaches. Beyond conformational flexibility, molecular recognition in living systems takes place in the presence of explicit solvent molecules and physiological ion conditions, which strongly influence interaction stability, hydrogen bonding networks, and electrostatic balance. Therefore, to better approximate the natural aqueous environment and to capture time-dependent structural adaptations of the complexes, MD simulations were performed. These simulations enabled the evaluation of the stability, persistence, and dynamic evolution of ligand–protein interactions over time.

AKT1 and mTOR were selected for focused MD analysis because they represent central downstream components of the PI3Kα/AKT/mTOR survival-signaling axis and showed favorable predicted interactions with umbelliferone in the initial docking analysis. The MD simulations were performed to evaluate whether the docking-predicted interactions of umbelliferone with these selected signaling proteins remained stable under dynamic, explicit-solvent conditions rather than to comparatively rank all investigated targets. RMSD is a commonly used indicator of structural stability in MD simulations and is generally expected to remain within the 1 Å–3 Å range for stable protein–ligand complexes [25]. To ensure reproducibility, MD simulations were performed in triplicate for each system. RMSD values were calculated by aligning the protein backbone, while ligand deviations were computed relative to the binding site (Figure 2). For the AKT1–umbelliferone system, all three trajectories demonstrated rapid equilibration within the early phase of the simulation and remained stable throughout the 100 ns production period. The average RMSD values of the complexes ranged between 1.74 Å and 1.82 Å, with relatively small standard deviations (0.19 Å–0.36 Å), indicating a well-converged and dynamically stable interaction. In contrast, the ligand alone exhibited higher mobility, with mean RMSD values between 2.50 Å and 2.70 Å. A similar trend was observed for the mTOR–umbelliferone system. The complexes maintained structural stability across independent runs, yielding average RMSD values between 2.01 Å and 2.15 Å (SD: 0.22 Å–0.31 Å). Although slightly higher than AKT1, these values are consistent with persistent ligand accommodation within the binding pocket during the simulation time. Overall, the close agreement among independent trajectories supports the robustness of the simulations and the stability of umbelliferone binding.

Root-mean-square fluctuation (RMSF) analysis is commonly used to assess residue-level mobility, with higher RMSF values generally indicating regions of increased local flexibility [26]. Beyond distinguishing flexible from stable regions, RMSF analysis also enables the identification of key residues that may be essential for ligand interaction and overall receptor activity.

Residue-level flexibility of the complexes was evaluated using RMSF analysis across triplicate MD simulations (Figure 3). For the AKT1–umbelliferone system, the three independent trajectories yielded highly consistent profiles, with mean RMSF values of 0.74 Å–0.75 Å and standard deviations of 0.24 Å–0.26 Å, indicating limited backbone mobility and overall structural rigidity of the complex. Distinct fluctuation peaks were reproducibly observed around ARG41, SER205, and ASP387. These residues coincide with interaction regions identified in docking and MD contact analyses (Table 1 and Table 3), suggesting that the observed fluctuations may be associated with local ligand–protein interactions. In contrast, the mTOR–umbelliferone complex exhibited higher residue mobility across runs, with mean RMSF values ranging between 1.42 Å and 1.44 Å (SD: 0.38 Å–0.40 Å). Pronounced and reproducible peaks were detected near ASN1899 and ASP2195, which correspond to residues participating in hydrogen-bond interactions during the simulations. Although the ligand remained accommodated within the binding pocket, the broader amplitude of fluctuations suggests a comparatively more flexible local environment than that observed for AKT1. Overall, the agreement among independent trajectories reinforces the robustness of the simulations and indicates that umbelliferone binding is associated with reduced residue mobility in AKT1 relative to mTOR.

The global compactness of the protein structures was further evaluated by monitoring the radius of gyration (R_g_) across three independent trajectories (Figure 4). For the AKT1–umbelliferone system, R_g_ values remained highly stable throughout the simulations, with mean values of 21.89 Å, 22.09 Å, and 22.19 Å for runs 1–3, respectively, and very small standard deviations (~0.10 Å). The close overlap among trajectories and the absence of systematic drift confirm that the overall fold of AKT1 was well preserved upon ligand binding. The low dispersion among replicates further supports the convergence and reproducibility of the simulations. For the mTOR–umbelliferone complex, R_g_ values also demonstrated a highly stable behavior across the three independent simulations. The mean R_g_ values were 23.01 Å, 23.01 Å, and 23.01 Å, with standard deviations of approximately 0.22 Å for runs 1–3. The near-identical averages and minimal dispersion among trajectories indicate excellent reproducibility and confirm that ligand binding did not induce noticeable global conformational changes in the protein structure. Similar to AKT1, the absence of long-term drift or progressive expansion suggests that the mTOR fold remained compact and equilibrated throughout the 100 ns simulation period. The small fluctuations observed are consistent with natural thermal motions rather than structural instability.

Hydrogen-bond occupancy analysis was performed to evaluate the persistence of ligand–protein interactions throughout the MD simulations. Hydrogen bonds represent important noncovalent interactions that can contribute to the stabilization of protein–ligand complexes [27]. In this context, occupancy represents the percentage of analyzed trajectory frames in which a specific hydrogen bond is present, with higher occupancy values indicating greater persistence of the interaction during the simulation [28]. To focus the analysis on the more persistent hydrogen-bonding interactions, only hydrogen bonds with occupancy values greater than 10% were considered in the revised analysis.

Among the investigated complexes, AKT1–umbelliferone exhibited a prominent hydrogen bond involving SER205 (main chain), with an occupancy of 49.10% (Table 3), indicating that this interaction was maintained across a substantial proportion of the analyzed trajectory. Notably, SER205 was also identified as an interacting residue in the molecular docking analysis, providing consistency between the docking-predicted binding mode and the interaction pattern observed under dynamic conditions. In contrast, no individual hydrogen bond in the mTOR–umbelliferone complex exhibited an occupancy greater than 10%, indicating a more transient hydrogen-bonding pattern within the analyzed trajectory. Overall, the hydrogen-bond occupancy analysis revealed a more persistent ligand–protein hydrogen-bond interaction for the AKT1–umbelliferone complex than for the mTOR–umbelliferone complex, consistent with the comparatively favorable interaction profile observed for AKT1 in the complementary computational analyses.

MM/PBSA calculations were conducted to further estimate the interaction energetics of umbelliferone in complex with AKT1 and mTOR (Table 4). MM/PBSA calculations were performed using 100 snapshots extracted from the equilibrated segment of the production trajectories (frames 900–999 inclusive; one snapshot per frame). Among the analyzed targets, umbelliferone showed a more favorable MM/PBSA energetic estimate for AKT1 (−3.73 ± 8.05 kcal/mol) compared with mTOR (−1.11 ± 6.90 kcal/mol), suggesting a comparatively greater stabilization of the AKT1−umbelliferone complex. For AKT1, the calculated MM/PBSA energetic estimate reflected the combined contributions of multiple energetic components, with nonpolar interactions representing the primary stabilizing factor, while electrostatic contributions were largely offset by polar solvation effects. In contrast, the umbelliferone–mTOR complex exhibited weaker overall stabilization, as indicated by a less favorable MM/PBSA energetic estimate despite comparable nonpolar interaction terms. Overall, these findings point to a preferential predicted binding of umbelliferone toward AKT1 relative to mTOR within the MM/PBSA framework. The relatively large uncertainty observed for the Poisson–Boltzmann term is not unexpected, as $∆G_{Poisson-Boltzmann}$ is typically the most fluctuation-prone component in MM/PBSA due to its sensitivity to instantaneous charge distributions, solvent rearrangements, and local dielectric boundary changes. Therefore, the MM/PBSA results were interpreted comparatively (AKT1 vs. mTOR) rather than as complete Gibbs binding free energies.

To assess the temporal stability of the MM/PBSA estimates, an additional time-window analysis was performed over five consecutive 10 ns intervals between 50 and 100 ns (Table S1). For the mTOR–umbelliferone complex, the mean MM/PBSA energetic estimates remained highly consistent across the analyzed intervals, with no apparent systematic temporal drift. In contrast, the AKT1–umbelliferone complex showed a gradual shift toward more favorable mean energies during the earlier intervals, followed by comparable estimates during the final 20 ns, indicating stabilization of the mean energetic estimate toward the end of the trajectory. Nevertheless, the relatively large standard deviations observed within individual time windows for both complexes indicate substantial energetic fluctuations and warrant cautious interpretation of the MM/PBSA estimates.

### 2.3. ADME Results

The in silico physicochemical, drug-likeness, and pharmacokinetic properties of umbelliferone were evaluated using the SwissADME web-based platform. Umbelliferone showed a predicted drug-like small-molecule profile, with a molecular weight of 162.14 g/mol, no rotatable bonds, and a topological polar surface area (TPSA) of 50.44 Å^2^. The molecule contains one hydrogen-bond donor and three hydrogen-bond acceptors and showed no violations of Lipinski’s rule of five. Umbelliferone also satisfied the Veber and Egan criteria, while one violation of the Muegge filter was predicted due to its molecular weight being below 200 g/mol. No PAINS alerts were identified in the computational screening.

The consensus logP value was predicted to be 1.51, indicating moderate lipophilicity, while the estimated aqueous solubility (ESOL logS = −2.46) classified umbelliferone as soluble. SwissADME further predicted high gastrointestinal absorption and no blood–brain barrier permeation. These parameters represent computational estimates of physicochemical and ADME-related properties and should not be interpreted as experimentally determined absorption, distribution, or pharmacokinetic behavior.

Overall, the in silico analysis indicates that umbelliferone satisfies several commonly used drug-likeness criteria and exhibits predicted physicochemical and ADME characteristics of potential relevance to further pharmacological investigation. However, these predictions require validation through appropriate experimental pharmacokinetic and in vivo studies.

### 2.4. Effect of Umbelliferone on Cell Viability

Umbelliferone treatment induced a concentration-dependent reduction in the viability of MCF-7 breast cancer cells after 24 h of exposure (Figure 5A). The half-maximal inhibitory concentration (IC_50_) was determined to be 492 µM. Compared with the untreated control, the reduction in cell viability was statistically significant at concentrations of 50 µM and above, with Dunn’s multiple-comparison adjusted *p* values of 0.0437, 0.0121, <0.0001, and <0.0001 for 50, 100, 200, and 400 µM umbelliferone, respectively. In contrast, the effect observed at 25 µM was not statistically significant (adjusted *p* = 0.797).

Umbelliferone exhibited substantially lower cytotoxicity toward BJ normal fibroblasts than toward MCF-7 cells. Because BJ cell viability did not decrease to 50% within the experimentally tested concentration range (up to 1400 µM), an experimentally defined IC_50_ could not be determined, and the BJ IC_50_ was therefore reported as >1400 µM. Accordingly, based on the MCF-7 IC_50_ value of 492 µM, the selectivity index (SI), calculated as IC_50_(BJ)/IC_50_(MCF-7), was >2.85. Cisplatin (40 µg/mL; ~133 µM), included as a positive control, markedly reduced cell viability to 20.12% in MCF-7 cells and 15.73% in BJ fibroblasts, confirming the responsiveness of the assay system.

### 2.5. Effect of Umbelliferone on Apoptosis

To determine whether the observed cytotoxic effects were associated with apoptosis, Annexin V-FITC/PI staining followed by flow cytometric analysis was performed. As shown in Figure 6, umbelliferone treatment increased the apoptotic cell population in MCF-7 cells compared with the untreated control. The total apoptosis rate (early + late apoptosis) increased from 10.57% in the control group to 22.21% and 25.55% following treatment with 400 and 800 µM umbelliferone, respectively. Although an increase in total apoptosis was observed at 400 µM, the difference did not reach statistical significance compared with the untreated control (Dunn’s adjusted *p* = 0.0731). In contrast, treatment with 800 µM umbelliferone resulted in a statistically significant increase in total apoptosis (Dunn’s adjusted *p* = 0.0122). Both early and late apoptotic populations were elevated following umbelliferone treatment, supporting an association between the reduction in cell viability and apoptosis induction in MCF-7 cells.

### 2.6. Effect of Umbelliferone on Colony Formation

The long-term effect of umbelliferone on the clonogenic potential of MCF-7 cells was evaluated using a colony formation assay. Representative crystal violet-stained images showed extensive colony formation in untreated control cells, whereas reduced colony formation was visually observed following treatment with umbelliferone and cisplatin (Figure 7). Individual colonies in the untreated control group could not be reliably quantified because of extensive colony merging and were therefore reported as not determined (ND). Among the quantifiable treatment groups, the mean numbers of countable colonies were 7.7 ± 0.6 and 6.0 ± 0.0 following treatment with 400 and 800 µM umbelliferone, respectively, corresponding to colony-forming efficiencies (CFEs) of 15.3 ± 1.2% and 12.0 ± 0.0%. Cisplatin treatment resulted in the lowest number of countable colonies (2.7 ± 1.2), with a CFE of 5.3 ± 2.3% (Table 5). These findings are consistent with reduced clonogenic survival following umbelliferone treatment; however, because individual colonies in the untreated control group could not be reliably quantified, a quantitative treatment-versus-control comparison based on colony counts was not performed.

### 2.7. Effect of Umbelliferone on Caspase Activity

Caspase-3/7 and Caspase-9 activities were evaluated to further characterize apoptosis-associated responses following umbelliferone treatment (Figure 8). Umbelliferone at 400 µM significantly increased relative Caspase-3/7 activity to 1.873 ± 0.635 compared with the untreated control (*p* = 0.027), whereas no significant increase was observed at 800 µM (1.096 ± 0.100; *p* = 0.270). Similarly, relative Caspase-9 activity was significantly increased at 400 µM (2.483 ± 0.092; *p* = 0.014), but not at 800 µM (1.040 ± 0.122; *p* = 0.891). In addition, Caspase-3/7 and Caspase-9 activities at 400 µM were significantly higher than those observed at 800 µM (*p* = 0.034 and *p* = 0.021, respectively). These findings indicate that caspase activation was most pronounced at 400 µM under the experimental conditions employed and was not sustained at the higher umbelliferone concentration.

## 3. Discussion

In the present study, the biological effects of umbelliferone in MCF-7 breast cancer cells were investigated through an integrated in silico and in vitro approach, together with computational evaluation of its potential interactions with proteins associated with cellular survival and apoptotic signaling. The MTT assay demonstrated that umbelliferone reduced MCF-7 cell viability in a concentration-dependent manner following 24 h of treatment, with an IC_50_ value of 492 µM. Although this value indicates moderate cytotoxic potency, it is comparable to that reported by Mousavi et al. [22], who observed an IC_50_ value of 476.3 µM in MCF-7 cells under different experimental conditions. In contrast, other studies have reported markedly different cytotoxic responses to umbelliferone in breast cancer models [21,29]. Such discrepancies are likely attributable to variations in experimental parameters, including cell type, treatment duration, compound purity, assay conditions, and experimental design. Therefore, direct comparison of IC_50_ values among different studies should be interpreted with caution.

Importantly, the biological activity of natural compounds should not be evaluated solely on the basis of IC_50_ values. Many naturally occurring coumarins exhibit moderate cytotoxic potency [30] while simultaneously modulating multiple cellular processes, including apoptosis, oxidative stress, and cell proliferation. Moreover, the MTT assay primarily reflects mitochondrial metabolic activity rather than direct cell death [31]. Consequently, compounds possessing antioxidant or redox-modulating properties, such as umbelliferone, may partially preserve mitochondrial function while simultaneously activating downstream apoptotic pathways, thereby influencing metabolic readouts [32,33]. For this reason, complementary functional assays are essential to obtain a more comprehensive assessment of their biological effects.

Accordingly, the present study combined cell viability analysis with apoptosis, caspase activity, clonogenic survival, and selectivity assessments. Annexin V-FITC/PI flow cytometry showed an increase in apoptotic cell populations following umbelliferone treatment, with a statistically significant increase in total apoptosis observed at 800 µM. Previous studies have also reported apoptosis-associated effects of umbelliferone in breast cancer models. Notably, however, although Rashmi et al. [21] evaluated the cytotoxic activity of umbelliferone in both MCF-7 and MDA-MB-231 cells, their Annexin V-FITC/PI apoptosis analysis was performed in MDA-MB-231 cells. Thus, the present findings extend these previous observations by demonstrating an apoptosis-associated response to umbelliferone specifically in MCF-7 cells using Annexin V-FITC/PI flow cytometry.

To further characterize apoptosis-associated responses, Caspase-3/7 and Caspase-9 activities were evaluated. At 400 µM, umbelliferone significantly increased relative Caspase-3/7 and Caspase-9 activities to 1.873 ± 0.635 and 2.483 ± 0.092, respectively. In contrast, neither Caspase-3/7 nor Caspase-9 activity was significantly increased at 800 µM. Because MCF-7 cells are deficient in functional Caspase-3, the Caspase-3/7 assay signal should be interpreted primarily in the context of Caspase-7-associated activity. The concomitant increase in Caspase-3/7 and Caspase-9 activities at 400 µM provides complementary biochemical evidence consistent with caspase-associated apoptotic signaling under this treatment condition. However, the absence of increased caspase activity at 800 µM, despite the significant increase in total apoptosis detected by Annexin V-FITC/PI at this concentration, indicates that the apoptotic and caspase activity profiles did not follow a simple concentration-dependent relationship. This difference may reflect temporal differences in caspase activation or the contribution of additional cell-death processes at the higher concentration; however, the present data do not allow these possibilities to be distinguished. Further time-course and mechanistic studies would therefore be required to clarify the basis of this non-monotonic caspase response.

Although the MTT assay reflects short-term metabolic changes, the colony formation assay evaluates the long-term reproductive capacity of surviving cells. The colony formation assay provided additional evidence suggesting that the effects of umbelliferone extended beyond short-term changes in metabolic viability. Representative images showed reduced clonogenic growth following umbelliferone treatment, while quantification of the countable treatment groups yielded CFEs of 15.3 ± 1.2% and 12.0 ± 0.0% at 400 and 800 µM, respectively. However, because extensive colony merging prevented reliable quantification of individual colonies in the untreated control group, these findings should be interpreted as supportive rather than definitive quantitative evidence of reduced clonogenic survival.

Overall, these findings indicate that the biological effects of umbelliferone in MCF-7 cells cannot be interpreted solely on the basis of its IC_50_ value. Instead, the combined evidence from cell viability, apoptosis, caspase activity, clonogenic survival, selectivity analysis, and computational modeling provides a broader characterization of its biological activity and supports further investigation of umbelliferone in breast cancer models.

To place the docking results in a comparative context, the predicted binding scores of umbelliferone were evaluated against both the corresponding co-crystallized reference ligands and established inhibitors of the investigated targets using the same AutoDock Vina protocol. The established inhibitors capivasertib, AZD8055, and alpelisib exhibited more favorable predicted docking scores toward AKT1, mTOR, and PI3Kα, respectively, than umbelliferone. In contrast, the predicted docking scores of umbelliferone and Ac-DNLD-CHO toward Caspase-7 were similar, with umbelliferone yielding a slightly more favorable score (−6.91 vs. −6.70 kcal/mol). Given the small difference between these computational estimates, this finding should be interpreted cautiously and does not imply that umbelliferone possesses greater affinity or inhibitory activity toward Caspase-7. Nevertheless, umbelliferone showed predicted interactions within the corresponding binding pockets, particularly with AKT1, for which the difference relative to capivasertib was comparatively modest. Importantly, docking scores represent computational estimates of ligand–protein interactions and cannot be considered equivalent to experimentally determined binding affinities or evidence of target inhibition. Therefore, the present docking results are interpreted as providing computational hypotheses regarding potential interactions of umbelliferone with these proteins rather than demonstrating direct modulation of their activity.

The MD analyses provided additional insight into the structural behavior of the complexes beyond static docking predictions. RMSD trajectories obtained from triplicate simulations indicated that both the AKT1–umbelliferone and mTOR–umbelliferone complexes reached relatively stable conformational regimes and maintained consistent behavior throughout the 100 ns production runs. The relatively small deviations and close agreement among independent replicas support the reproducibility of ligand accommodation within the binding pockets.

Residue-level flexibility analysis further revealed that the AKT1 complex exhibited overall lower fluctuations compared with mTOR, suggesting a more rigid local environment upon ligand binding. Regions displaying moderate mobility coincided with residues involved in ligand interactions, indicating that limited flexibility may contribute to adaptive stabilization rather than disrupt binding.

In addition, R_g_ profiles remained highly consistent across triplicate simulations for both systems, indicating that global protein compactness was preserved in the presence of umbelliferone. Together, these observations support the dynamic stability of the complexes under explicit solvent conditions and provide a structural basis for interpreting the MM/PBSA energetic analyses.

MM/PBSA energetic estimates indicated a comparatively more favorable interaction of umbelliferone with AKT1 than with mTOR. Together with the slightly more favorable docking score for AKT1 and the reproducible behavior observed across the MD simulations, these findings suggest a preferential stabilization of the AKT1–umbelliferone complex under the computational conditions employed. Although the biological significance of this interaction requires further experimental investigation, the computational findings identify AKT1 as a potentially relevant protein for future mechanistic studies.

Relatively large standard deviations were observed in the MM/PBSA energy components, indicating substantial energetic fluctuations among the sampled conformations and limiting interpretation of the calculated values as precise quantitative estimates of interaction energetics. To further evaluate the temporal stability of these estimates, MM/PBSA calculations were compared across five consecutive 10 ns intervals between 50 and 100 ns. The mTOR–umbelliferone complex showed highly consistent mean energy estimates across the analyzed intervals, with no apparent systematic temporal drift, whereas the AKT1–umbelliferone complex exhibited a progressive shift toward more favorable mean energies followed by stabilization during the final 20 ns. Importantly, temporal stability of the MM/PBSA estimate should be distinguished from the magnitude of the MM/PBSA energetic estimate; thus, the more stable time-window profile observed for mTOR does not imply a more favorable predicted interaction than that observed for AKT1. Such energetic variability may arise from conformational sampling, solvation effects, and approximations inherent to continuum-solvent models. Accordingly, the MM/PBSA results were interpreted primarily as comparative estimates of the relative interaction tendencies of the investigated complexes rather than as experimentally equivalent absolute binding affinities [34,35].

The comparatively weaker interaction predicted for the umbelliferone–mTOR complex may be related to the structural complexity and size of the mTOR binding region, which can influence ligand accommodation and solvation effects. Nevertheless, the persistence of ligand binding throughout the simulations suggests that both proteins remain relevant candidates for future experimental investigation.

From a broader pharmacological perspective, the predicted interaction profile is compatible with the multi-target behavior reported for many natural products, which may interact with multiple components of cellular signaling networks rather than acting as highly selective, high-affinity single-target inhibitors [36,37,38]. However, computational prediction of interactions with multiple proteins does not establish functional modulation of these targets, and experimental validation is required to determine their biological relevance.

The in silico ADME analysis provided additional information regarding the predicted physicochemical and drug-likeness characteristics of umbelliferone. SwissADME predictions indicated compliance with several commonly applied drug-likeness criteria, together with predicted high gastrointestinal absorption and no blood–brain barrier permeation. However, these findings represent computational estimates and should not be interpreted as experimentally established pharmacokinetic behavior. In particular, in silico predictions cannot account fully for factors such as formulation-dependent absorption, metabolic stability, systemic exposure, tissue distribution, or clearance under physiological conditions. Therefore, the ADME results should be regarded as preliminary supportive information for future pharmacological investigation, and experimental pharmacokinetic and in vivo studies will be required to determine the actual absorption, distribution, metabolism, and elimination profile of umbelliferone.

Taken together, the in vitro findings indicate that umbelliferone reduces MCF-7 cell viability, promotes apoptotic cell death, and impairs clonogenic survival while exhibiting comparatively lower cytotoxicity toward BJ normal fibroblasts. Complementary computational analyses predicted interactions with proteins associated with cellular survival and apoptotic signaling, with AKT1 exhibiting the most favorable overall interaction profile among the targets subjected to detailed dynamic analysis. These computational observations should nevertheless be regarded as hypothesis-generating and require experimental target-level validation.

A limitation of the present study is that, although apoptosis induction and caspase activity were experimentally evaluated, the upstream molecular events responsible for these effects were not directly investigated. Moreover, the different concentration–response profiles observed for Annexin V-FITC/PI positivity and caspase activity cannot be mechanistically resolved from the present single-time-point measurements. Time-course studies would be valuable for determining the temporal relationship between caspase activation and the development of apoptotic cell populations. In addition, the computationally predicted interactions with PI3Kα, AKT1, and mTOR were not experimentally validated at the protein-expression, phosphorylation, or direct target-engagement level. Future studies incorporating pathway-specific approaches, including analysis of PI3K expression and AKT/mTOR phosphorylation status, as well as direct target-validation strategies, would therefore be valuable for determining whether the computationally predicted interactions contribute to the observed biological effects. Accordingly, the present computational findings should be regarded as hypothesis-generating evidence rather than definitive demonstration of PI3Kα/AKT/mTOR pathway modulation.

## 4. Materials and Methods

### 4.1. Molecular Docking

In this study, molecular docking analysis was performed to investigate the potential interactions of umbelliferone with AKT1, mTOR, PI3Kα, and Caspase-7, proteins associated with cellular survival and apoptotic processes in breast cancer. The three-dimensional crystal structures of AKT1 (PDB ID: 4EJN), mTOR (PDB ID: 4JSV), PI3Kα (PDB ID: 4L23), and Caspase-7 (PDB ID: 2QLF) were obtained from the RCSB Protein Data Bank. Water molecules and co-crystallized ligands were removed from the protein structures, and polar hydrogen atoms and Kollman charges were added using Discovery Studio Visualizer, version 2025 (Accelrys Software Inc., San Diego, CA, USA) and AutoDockTools 1.5.7 (The Scripps Research Institute, La Jolla, CA, USA). Missing residues in the target protein structures were completed using Swiss-PdbViewer, version 4.10 (SPDBV; Swiss Institute of Bioinformatics, Lausanne, Switzerland) [39].

The three-dimensional structure of umbelliferone (PubChem CID: 5281426) was obtained from the PubChem database and geometrically optimized using Gaussian 09 (Gaussian, Inc., Wallingford, CT, USA) [40] at the B3LYP/6-31G(d,p) level of density functional theory [41,42]. Molecular docking calculations were subsequently performed using AutoDock Vina (version 1.2.7) (Center for Computational Structural Biology, The Scripps Research Institute, La Jolla, CA, USA) [43]. The binding sites were defined based on the positions of the corresponding co-crystallized ligands. For each target, a grid box of 80 × 80 × 80 Å with spacing 0.375 Å was centered at (31.755, 41.230, 13.624) for AKT1, (−5.379, −33.126, −53.055) for mTOR, (18.095, 34.177, 30.198) for PI3Kα, and (−48.273, 16.978, 32.882) for Caspase-7. The exhaustiveness value was set to 8, up to nine binding poses were generated for each docking run, and the energy range was set to 4 kcal/mol. The first-ranked pose, corresponding to the lowest predicted binding energy within the predefined binding pocket, was selected for subsequent interaction analysis. Protein–ligand complexes were visualized and analyzed using Discovery Studio Visualizer.

To provide a methodologically consistent reference for the predicted docking scores of umbelliferone, the corresponding co-crystallized ligands were also docked using the same AutoDock Vina protocol. OR4, ADP, X6K, and Ac-DNLD-CHO were used as the co-crystallized reference ligands for AKT1, mTOR, PI3Kα, and Caspase-7, respectively. In addition, established target-directed inhibitors were selected to provide a pharmacologically relevant benchmark. Capivasertib (AZD5363), an ATP-competitive AKT inhibitor [44], AZD8055, an ATP-competitive mTOR kinase inhibitor [45], and alpelisib (NVP-BYL719), a selective PI3Kα inhibitor [46], were selected as comparator compounds for AKT1, mTOR, and PI3Kα, respectively. For Caspase-7, Ac-DNLD-CHO was selected as the reference inhibitor based on its experimentally characterized interaction with Caspase-7 and its co-crystallized complex available in the 2QLF structure [47]. All comparator compounds were docked using the same AutoDock Vina parameters and binding-site definitions applied to umbelliferone to ensure methodological consistency.

As an independent validation of the docking protocol, the selected co-crystallized ligands were additionally re-docked into their respective crystallographic binding sites using AutoDock 4.2 (The Scripps Research Institute, La Jolla, CA, USA) [48]. The root-mean-square deviation (RMSD) between each re-docked pose and its experimentally determined crystallographic conformation was calculated. RMSD values below 2 Å were considered indicative of successful reproduction of the experimentally observed binding mode.

### 4.2. Molecular Dynamics

The conformational behavior of umbelliferone in complex with AKT1 and mTOR was further investigated using molecular dynamics (MD) simulations. AKT1 and mTOR were selected for this focused dynamic analysis because they represent central downstream components of the PI3Kα/AKT/mTOR survival-signaling axis and exhibited favorable predicted interactions with umbelliferone in the initial docking analysis. The purpose of the MD simulations was not to comparatively rank all investigated targets, but rather to determine whether the docking-predicted interactions of umbelliferone with these selected signaling proteins remained stable under dynamic, explicit-solvent conditions. PI3Kα, representing the upstream component of the signaling axis, and Caspase-7, included as an apoptosis-associated executioner caspase relevant to the MCF-7 cellular model, were evaluated at the molecular docking level but were not subjected to MD simulations.

MD simulations of the AKT1–umbelliferone and mTOR–umbelliferone complexes were performed using NAMD/VMD, version 3.0.2 (Theoretical and Computational Biophysics Group, University of Illinois at Urbana–Champaign, Urbana, IL, USA) [49,50] with the CHARMM22 force field [51,52]. The CHARMM22 force field was selected because it is a well-established all-atom force field for biomolecular simulations and provides a consistent framework for describing protein interactions. In addition, its compatibility with the CHARMM-formatted ligand parameters generated using SwissParam enabled a consistent parameterization of the protein–ligand systems. Topology and parameter files for umbelliferone were generated using the SwissParam web server (Molecular Modelling Group, University of Lausanne and SIB Swiss Institute of Bioinformatics, Lausanne, Switzerland) [53] and were directly employed in NAMD without additional modification. Following generation of the required topology and parameter files, the corresponding PDB–PSF pairs were constructed, and each protein–ligand complex was explicitly solvated using the TIP3P water model in a water box extending 10 Å beyond the solute in all directions. Na^+^ and Cl^−^ ions were subsequently added to neutralize the systems.

Energy minimization, heating, equilibration, and production simulations were subsequently performed according to the protocol adapted from Meshram’s Lab [54]. Following energy minimization, each system was gradually heated from 0 to 300 K over 300 ps using a 1 fs integration time step and subsequently equilibrated for 1 ns. Production simulations were conducted under isothermal–isobaric (NPT) conditions at 300 K and 1.01325 bar using a 2 fs integration time step. Temperature was controlled using Langevin dynamics with a damping coefficient of 1 ps^−1^, while pressure was maintained using the Langevin piston method with a piston period of 100 fs and a piston decay time of 50 fs. Bonds involving hydrogen atoms were constrained during the simulations. A switching function was applied from 8 Å, with a nonbonded cutoff distance of 12 Å and a pair-list distance of 13.5 Å. Long-range electrostatic interactions were treated using the particle mesh Ewald (PME) method with a tolerance of 1 × 10^−6^ and grid dimensions of 74 × 88 × 76 for AKT1 and 134 × 116 × 128 for mTOR.

For each replicate, a total production simulation time of 100 ns was achieved through 20 consecutive 5 ns segments, each comprising 2,500,000 integration steps at a 2 fs time step. Production simulations were independently performed in triplicate for each protein–ligand complex, and the resulting trajectories were used for subsequent analyses. Structural stability and conformational behavior were evaluated through backbone root-mean-square deviation (RMSD), residue-level root-mean-square fluctuation (RMSF), radius of gyration (R_g_), and hydrogen-bond analyses. RMSD calculations were performed using the RMSD Trajectory Tool in VMD. To remove overall translational and rotational motions, each trajectory frame was first aligned to the protein backbone relative to the initial reference structure. Ligand RMSD was subsequently calculated with respect to the aligned protein frame. In addition, MM/PBSA calculations were performed to estimate the relative interaction energetics and further characterize the stability of the protein–ligand interactions.

The MM/PBSA energetic estimate was calculated as:

(1) $$∆G_{MM/PBSA}=∆E_{MM}+∆G_{sol}$$

The gas-phase molecular mechanics energy (${\Delta E}_{MM}$) and the polar solvation free energy (${\Delta G}_{sol}$) can be expressed as follows:

(2) $$∆E_{MM}=∆E_{internal}+∆E_{electrostatic}+∆E_{van\;der\;Waals}$$

(3) $$∆G_{sol}=∆G_{Poisson-Boltzmann}+∆G_{Surface\;Area}$$

The configurational entropy contribution (−TΔS) was not calculated in the present analysis. Therefore, the reported MM/PBSA values represent enthalpy-dominated energetic estimates rather than complete Gibbs binding free energies. MM/PBSA calculations were performed in VMD using the Calculation of Free Energy (CaFE) plugin [55]. Within the CaFE workflow, NAMD was used to calculate the molecular mechanics energy components, whereas the Adaptive Poisson–Boltzmann Solver (APBS), version 3.4.1, was employed to determine the polar solvation free-energy contribution [56]. For the primary MM/PBSA analysis, 100 snapshots corresponding to trajectory frames 900–999 (90–100 ns) were extracted at one-frame intervals and used for MM/PBSA energetic estimation. To assess the temporal stability of the MM/PBSA estimates, an additional time-window analysis was performed over five consecutive 10 ns intervals spanning 50–100 ns (50–60, 60–70, 70–80, 80–90, and 90–100 ns). MM/PBSA calculations were independently performed for each interval using the same computational protocol, and the resulting mean energetic estimates and standard deviations were compared across successive windows to evaluate the presence of systematic temporal drift and the stability of the energetic estimates toward the end of the simulations.

### 4.3. ADME

Physicochemical properties are considered to be indicators of how well a drug can penetrate membranes [57]. Therefore, it is crucial to evaluate these properties of a drug candidate. The simplified molecular-input line-entry system (SMILES) form of umbelliferone was obtained from PubChem database. It was uploaded to SwissADME web server [58] for the analysis of pharmacokinetic and drug-likeness properties of umbelliferone.

### 4.4. MTT Cell Viability Assay

The MCF-7 human breast cancer cell line and BJ normal human fibroblast cell line were obtained from the existing cell culture stocks maintained in the Biophysics Laboratory, Faculty of Medicine, Aydın Adnan Menderes University (Aydın, Türkiye). Human breast cancer cells (MCF-7) and normal human fibroblast cells (BJ) were cultured in RPMI-1640 medium (Cat. No. 11875093, GIBCO, Thermo Fisher Scientific, Waltham, MA, USA) supplemented with 10% fetal bovine serum (FBS) (Cat. No. FBS-12A, Capricorn Scientific GmbH, Ebsdorfergrund, Germany), 1% penicillin–streptomycin (Cat. No. PS-B, Capricorn Scientific), and 1% L-glutamine (Cat. No. GLN-B, Capricorn Scientific). Cells were maintained at 37 °C in a humidified atmosphere containing 5% CO_2_.

Cell viability was evaluated using the MTT assay (Invitrogen™, Thermo Fisher Scientific, Waltham, MA, USA; Cat. No. M6494). Cells were seeded into 24-well plates at a density of 1 × 10^5^ cells per well and allowed to adhere overnight. Subsequently, MCF-7 cells were treated with increasing concentrations of umbelliferone (25–400 µM), whereas BJ fibroblasts were evaluated over an extended concentration range of up to 1400 µM because of their comparatively lower sensitivity to umbelliferone. Cells were exposed to the indicated concentrations for 24 h. Vehicle-treated cells served as controls, and the final concentration of dimethyl sulfoxide (DMSO; Sigma-Aldrich, St. Louis, MO, USA) did not exceed 0.1% (*v*/*v*).

Cisplatin (Sigma-Aldrich, St. Louis, MO, USA) (40 µg/mL; approximately 133 µM) was selected as a reference compound due to its well-established clinical use and documented cytotoxic activity in breast cancer therapy [59], and it was included as a positive control under identical experimental conditions. Dose–response curves were analyzed by nonlinear regression using a four-parameter logistic (4PL) model implemented in GraphPad Prism 9. IC_50_ values were determined when the 50% viability level was reached within the experimentally tested concentration range. For BJ fibroblasts, in which 50% viability was not reached at concentrations up to 1400 µM, the IC_50_ was reported as >1400 µM rather than using the extrapolated 4PL estimate as an experimentally determined value.

Following treatment, the culture medium was removed and cells were gently washed with phosphate-buffered saline (PBS; Gibco, Thermo Fisher Scientific, Waltham, MA, USA). Fresh medium containing MTT solution (final concentration: 0.5 mg/mL) was added, and the plates were incubated for 1.5 h at 37 °C. The MTT solution was then discarded, and the resulting formazan crystals were dissolved in DMSO. Absorbance was measured at 570 nm using a microplate reader (Epoch, BioTek Instruments, Winooski, VT, USA). Cell viability was expressed as a percentage relative to the vehicle control and calculated according to the following formula:

$$\%Cell\;viability=\frac{{OD}_{Sample}-{OD}_{Blank}}{{OD}_{Control}-{OD}_{Blank}}\times 100\%$$

### 4.5. Annexin V/PI Apoptosis Assay

Apoptotic cell death was evaluated using Annexin V-FITC/propidium iodide (PI) double staining followed by flow cytometric analysis. Human breast cancer MCF-7 cells were seeded in 6-well plates and allowed to reach approximately 60–70% confluence. Cells were then treated with umbelliferone (Sigma-Aldrich, St. Louis, MO, USA; Cat. No. H24003) at concentrations of 400 and 800 µM for 24 h, based on the results of preliminary MTT assays.

Following treatment, both floating and adherent cells were carefully collected, washed twice with cold phosphate-buffered saline (PBS), and resuspended in Annexin V binding buffer. Cells were subsequently stained using an Annexin V-FITC/PI apoptosis detection kit (Elabscience, Houston, TX, USA; Cat. No. E-CK-A211) according to the manufacturer’s instructions and incubated for 15 min at room temperature in the dark.

Samples were analyzed using a NovoCyte Flow Cytometer System (Agilent Technologies, Santa Clara, CA, USA), and at least 10,000 events were recorded per sample. Cell populations were quantified by quadrant analysis to distinguish live (Annexin V^−^/PI^−^), early apoptotic (Annexin V^+^/PI^−^), late apoptotic (Annexin V^+^/PI^+^), and necrotic (Annexin V^−^/PI^+^) cells. The assay was performed as three independent biological experiments (*n* = 3), with technical replicates included for each experimental condition.

### 4.6. Selectivity Index (SI)

The selectivity index (SI) was calculated as the ratio of the IC_50_ value in normal BJ fibroblasts to that in MCF-7 breast cancer cells (SI = IC_50_,BJ/IC_50_,MCF-7). Because 50% viability was not reached in BJ cells within the experimentally tested concentration range (up to 1400 µM), the BJ IC_50_ was considered to be >1400 µM. Accordingly, using the experimentally determined MCF-7 IC_50_ value of 492 µM, the SI was expressed as a lower-bound estimate (SI > 2.85) rather than as an exact value. Higher SI values indicate comparatively greater cytotoxic selectivity toward cancer cells relative to normal cells.

### 4.7. Colony-Forming Assay

To evaluate the long-term effect of umbelliferone on the clonogenic potential of MCF-7 cells, a colony formation assay was performed. Based on the MTT assay results, MCF-7 cells were treated with 400 and 800 µM umbelliferone for 24 h, while cisplatin-treated and untreated cells served as positive and negative controls, respectively. Following treatment, cells were harvested by trypsinization, counted, and reseeded into 24-well plates at a density of 50 cells per well in fresh complete medium without umbelliferone or cisplatin. The cultures were maintained for 14 days to allow colony formation.

At the end of the incubation period, colonies were gently washed twice with phosphate-buffered saline (PBS), fixed with absolute methanol, and stained with 0.05% crystal violet. Colonies containing at least 50 cells were counted under an inverted microscope (CK40; Olympus Corporation, Tokyo, Japan). The assay was performed in three independent biological experiments (*n* = 3), with technical replicates included for each experimental condition.

Colony-forming efficiency (CFE) was calculated as:

CFE (%) = (number of colonies counted/number of cells seeded) × 100

### 4.8. Caspase Activity Assay

Caspase-3/7 and Caspase-9 activities were evaluated using colorimetric activity assay kits (Elabscience, Houston, TX, USA; Cat. No. E-CK-A383 and E-CK-A389, respectively) according to the manufacturer’s instructions. MCF-7 cells were treated with 400 and 800 µM umbelliferone for 24 h, while untreated cells served as the control group.

Following treatment, approximately 1 × 10^6^ cells from each experimental group were collected and lysed using pre-chilled cell lysis buffer. Cell lysates were centrifuged at 11,000× *g* for 10–15 min at 4 °C, and the resulting supernatants were collected for enzymatic activity measurements. Total protein concentrations of the lysates were determined using the Bradford assay, and equal amounts of total protein were used for each reaction to ensure normalization among experimental groups.

Caspase-3/7 activity was determined using the Ac-DEVD-pNA chromogenic substrate, whereas Caspase-9 activity was evaluated using the Ac-LEHD-pNA substrate. The corresponding reaction mixtures containing 2× Reaction Buffer and the appropriate chromogenic substrate were incubated at 37 °C according to the manufacturer’s protocol. Enzymatic cleavage of the substrates released p-nitroaniline (pNA), and absorbance was measured at 405 nm using a microplate reader.

Because MCF-7 cells are deficient in Caspase-3 expression, the activity detected using the Caspase-3/7 assay was interpreted predominantly as Caspase-7-associated executioner caspase activity. All caspase activity experiments were performed as three independent biological experiments (*n* = 3), with each biological experiment analyzed in technical triplicate.

### 4.9. Statistical Analysis

All in vitro experiments were performed as three independent biological experiments (*n* = 3), with each independent experiment conducted in technical triplicate. Technical replicates within each independent experiment were averaged, and the resulting values from the three independent biological experiments were used as the statistical units for subsequent analyses. Given the small number of independent biological replicates (*n* = 3), formal assessment of normality was not considered sufficiently informative; therefore, no assumption of normal distribution was made and non-parametric statistical methods were employed. Differences among multiple groups were evaluated using the Kruskal–Wallis test followed by Dunn’s multiple-comparison post hoc test. Statistical analyses were carried out using GraphPad Prism 9 (GraphPad Software, Inc., Boston, MA, USA). Data are presented as mean ± standard deviation (SD), and differences were considered statistically significant at *p* < 0.05.

## 5. Conclusions

This study provides an integrated in vitro and computational evaluation of the biological effects of umbelliferone in MCF-7 breast cancer cells. Umbelliferone reduced MCF-7 cell viability in a concentration-dependent manner, while comparatively lower cytotoxicity was observed in BJ normal fibroblasts, with an IC_50_ of >1400 µM and a selectivity index of >2.85. Annexin V-FITC/PI analysis demonstrated increased apoptotic cell populations, and colony formation analysis indicated reduced clonogenic capacity following umbelliferone treatment. In addition, Caspase-3/7 and Caspase-9 activities were significantly increased at 400 µM, providing complementary biochemical evidence consistent with caspase-associated apoptotic signaling under this treatment condition. Notably, these increases were not observed at 800 µM, indicating that the caspase activity response was not sustained at the higher concentration. Computational analyses predicted interactions of umbelliferone with AKT1, mTOR, PI3Kα, and Caspase-7, while molecular dynamics and MM/PBSA analyses further characterized the interactions with AKT1 and mTOR. In silico ADME analysis indicated predicted physicochemical and pharmacokinetic characteristics compatible with commonly used drug-likeness criteria. However, these computational findings remain hypothesis-generating and do not establish direct target engagement, pathway inhibition, or in vivo pharmacokinetic behavior. Overall, the findings support further investigation of the biological and pharmacological effects of umbelliferone in breast cancer models, particularly through mechanistic target-validation and appropriate in vivo studies.

## Figures and Tables

**Figure 1:**
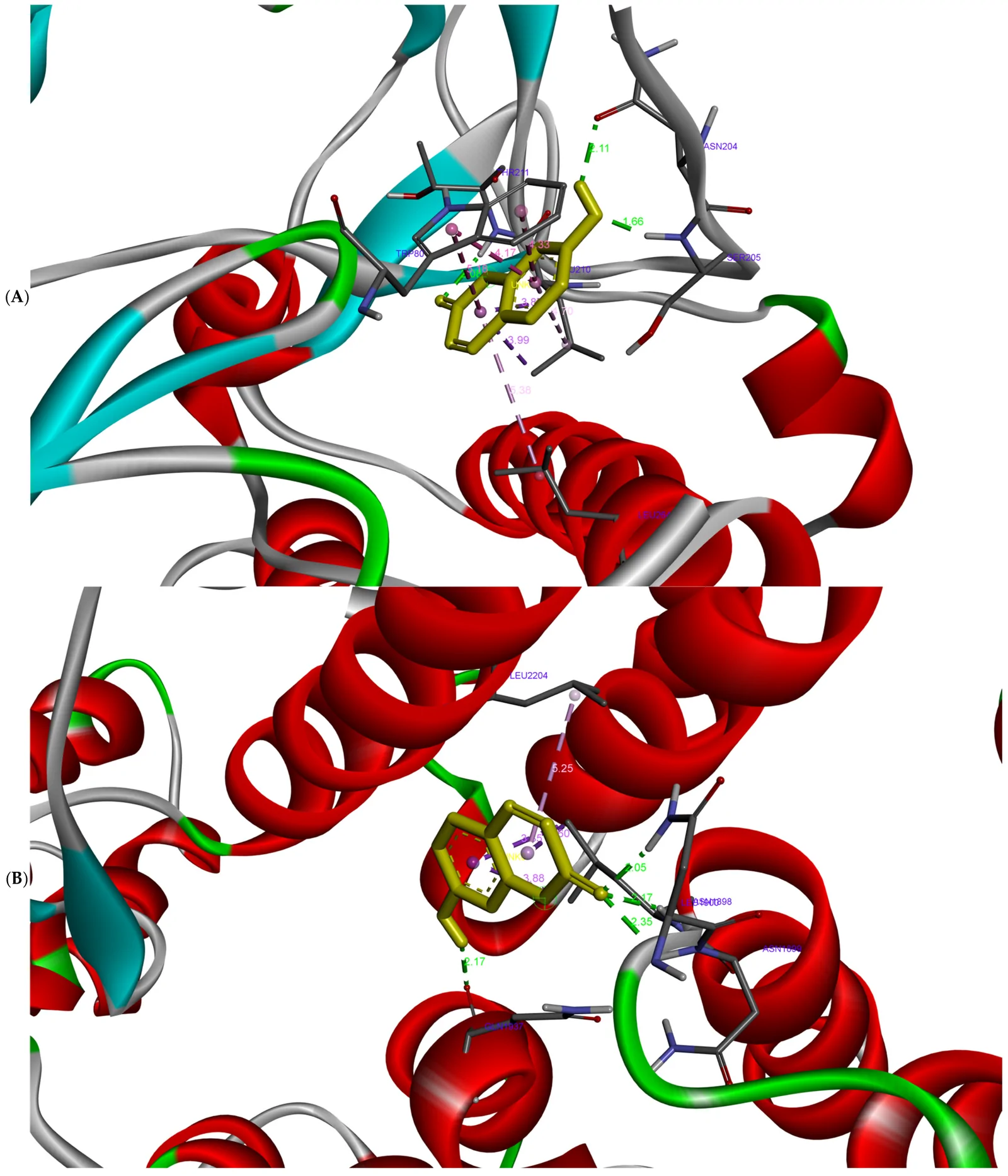

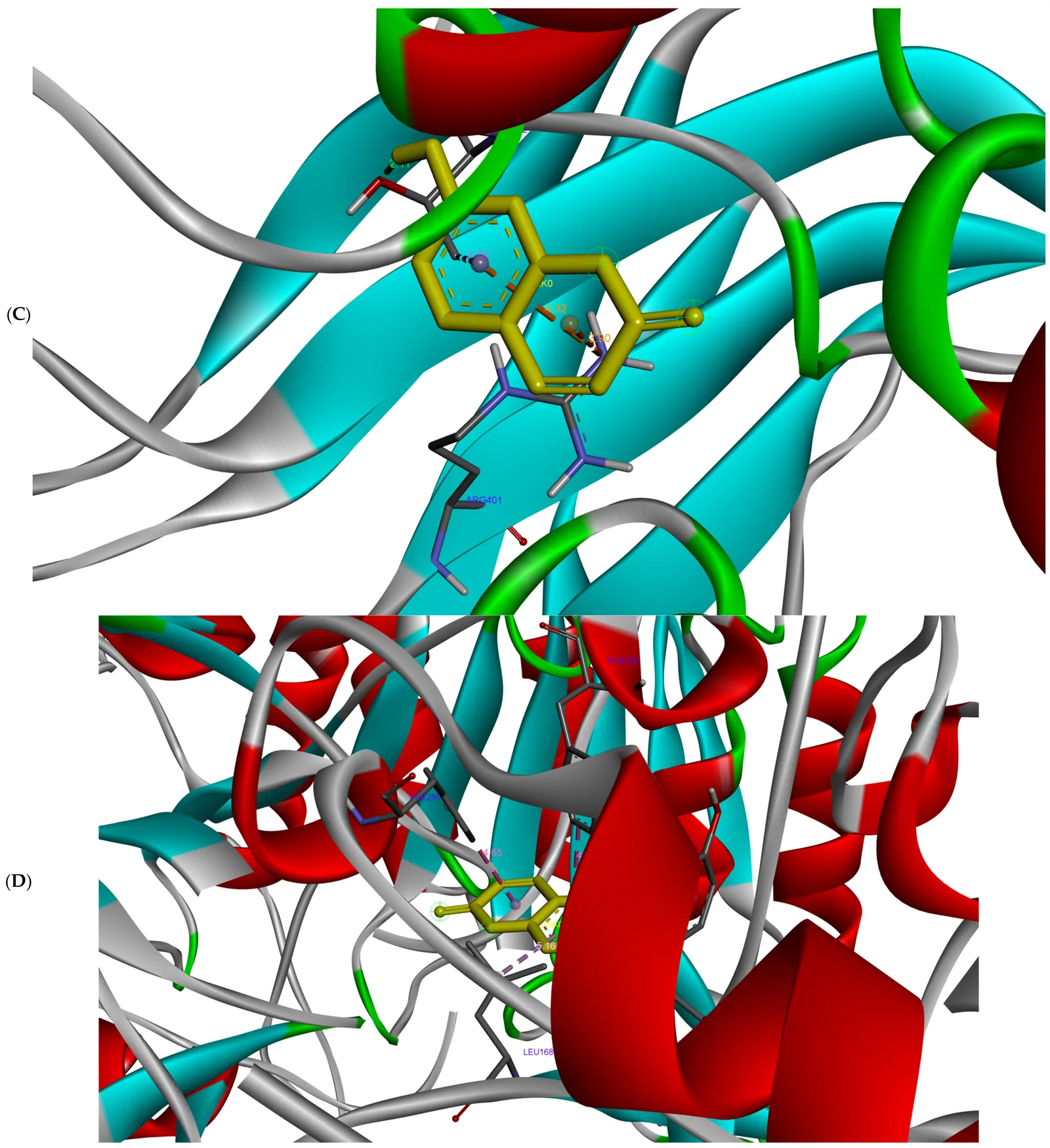
Predicted binding modes and molecular interactions of umbelliferone within the binding sites of (**A**) AKT1, (**B**) mTOR, (**C**) PI3Kα, and (**D**) Caspase-7 obtained by molecular docking using AutoDock Vina. The first-ranked docking pose, corresponding to the lowest predicted binding energy, is shown for each protein–ligand complex. Proteins are represented in cartoon format, while amino acid residues involved in ligand interactions are displayed as sticks. Umbelliferone is highlighted in yellow. Hydrogen-bonding and hydrophobic interactions are indicated by dashed lines.

**Figure 2:**
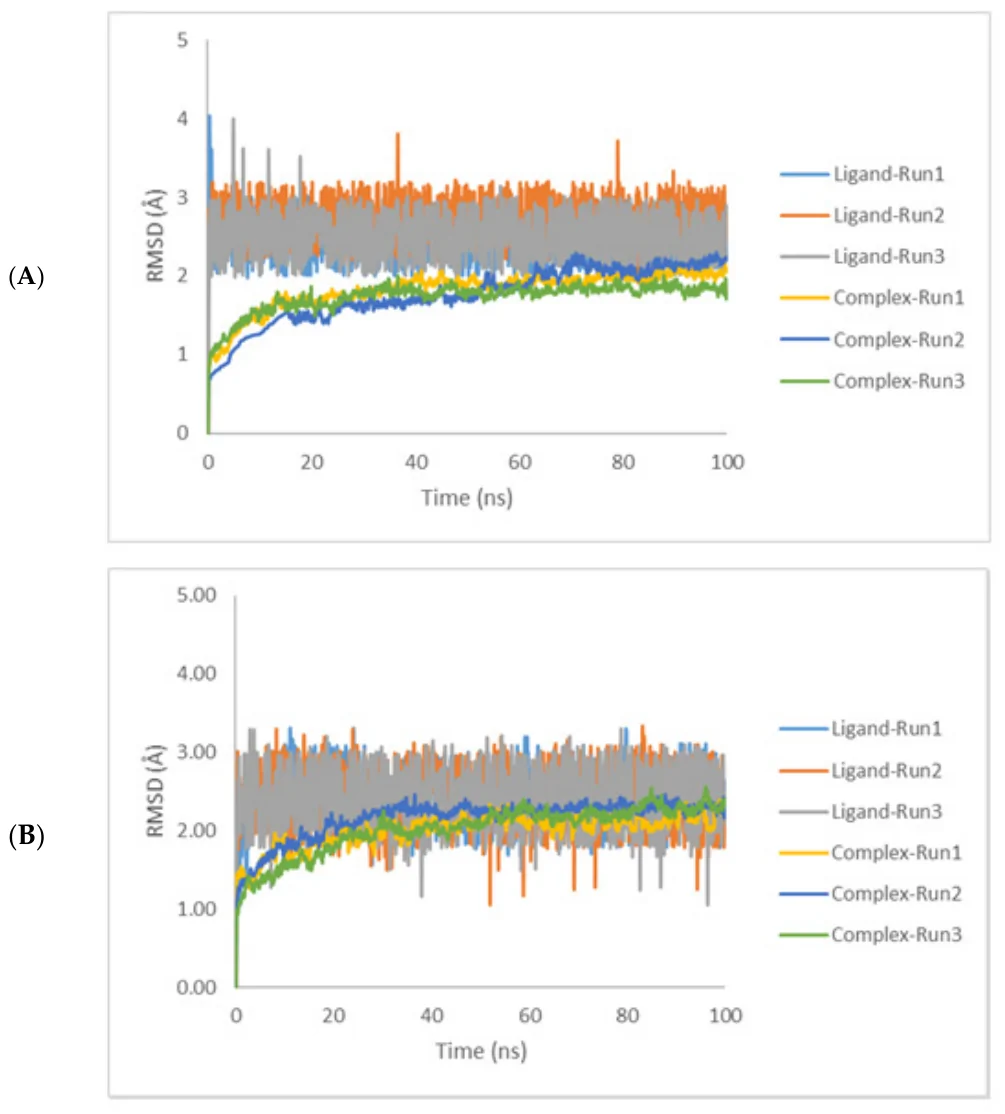
Root-mean-square deviation (RMSD) trajectories of the protein backbone and umbelliferone during 100 ns molecular dynamics simulations of the (**A**) AKT1–umbelliferone and (**B**) mTOR–umbelliferone complexes. Simulations were independently performed in triplicate for each complex. Prior to RMSD calculations, all trajectory frames were aligned to the protein backbone to remove overall translational and rotational motions. Ligand RMSD values were subsequently calculated relative to the aligned protein frame.

**Figure 3:**
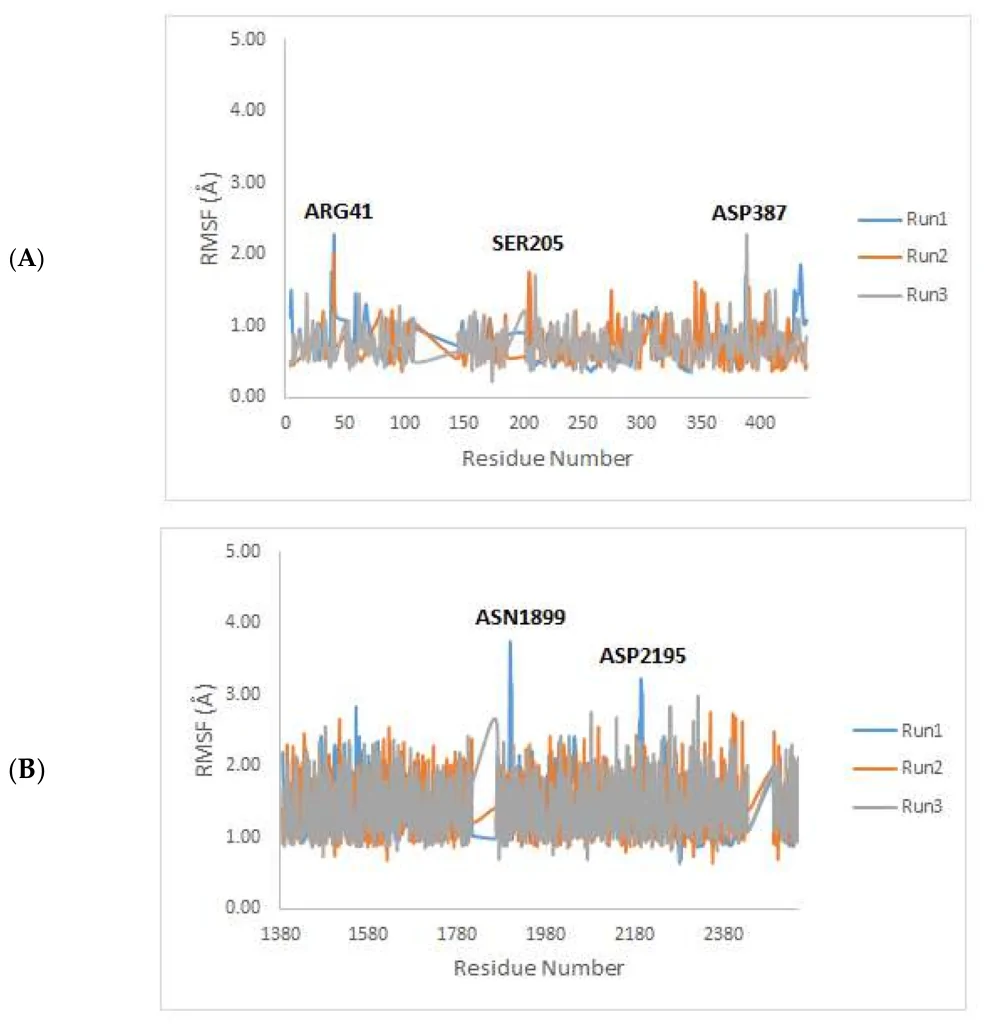
Residue-level root-mean-square fluctuation (RMSF) profiles of the (**A**) AKT1–umbelliferone and (**B**) mTOR–umbelliferone complexes obtained from three independent 100 ns molecular dynamics simulations. RMSF values represent the residue-wise conformational fluctuations observed during each independent trajectory, with higher values indicating regions of increased local flexibility.

**Figure 4:**
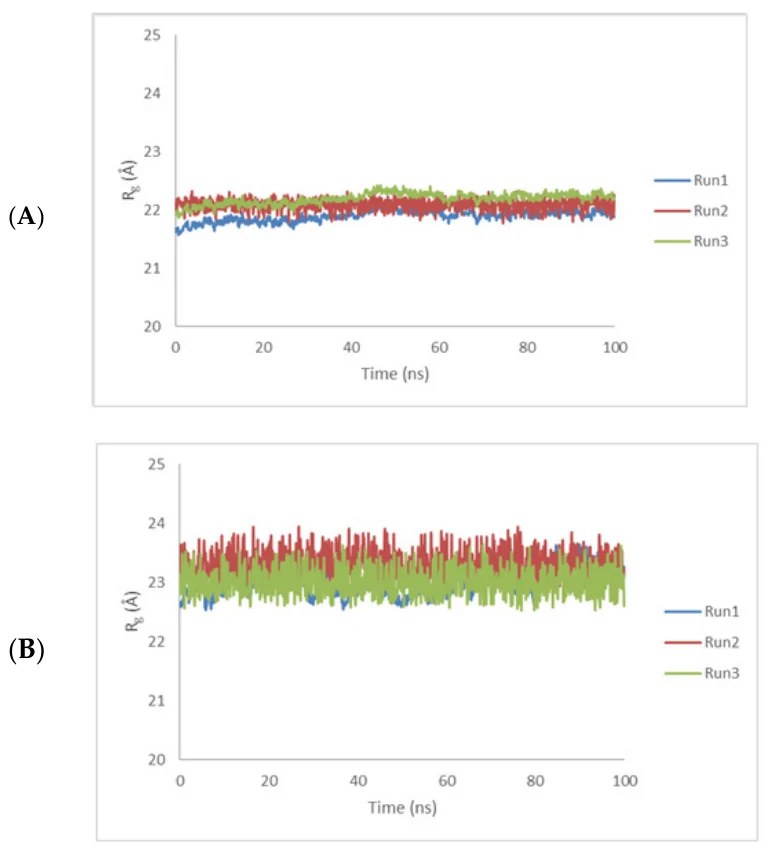
Radius of gyration (R_g_) profiles of the protein backbone for the (**A**) AKT1–umbelliferone and (**B**) mTOR–umbelliferone complexes obtained from three independent 100 ns molecular dynamics simulations. R_g_ values were monitored throughout each trajectory to evaluate changes in global protein compactness and structural organization during the simulations.

**Figure 5:**
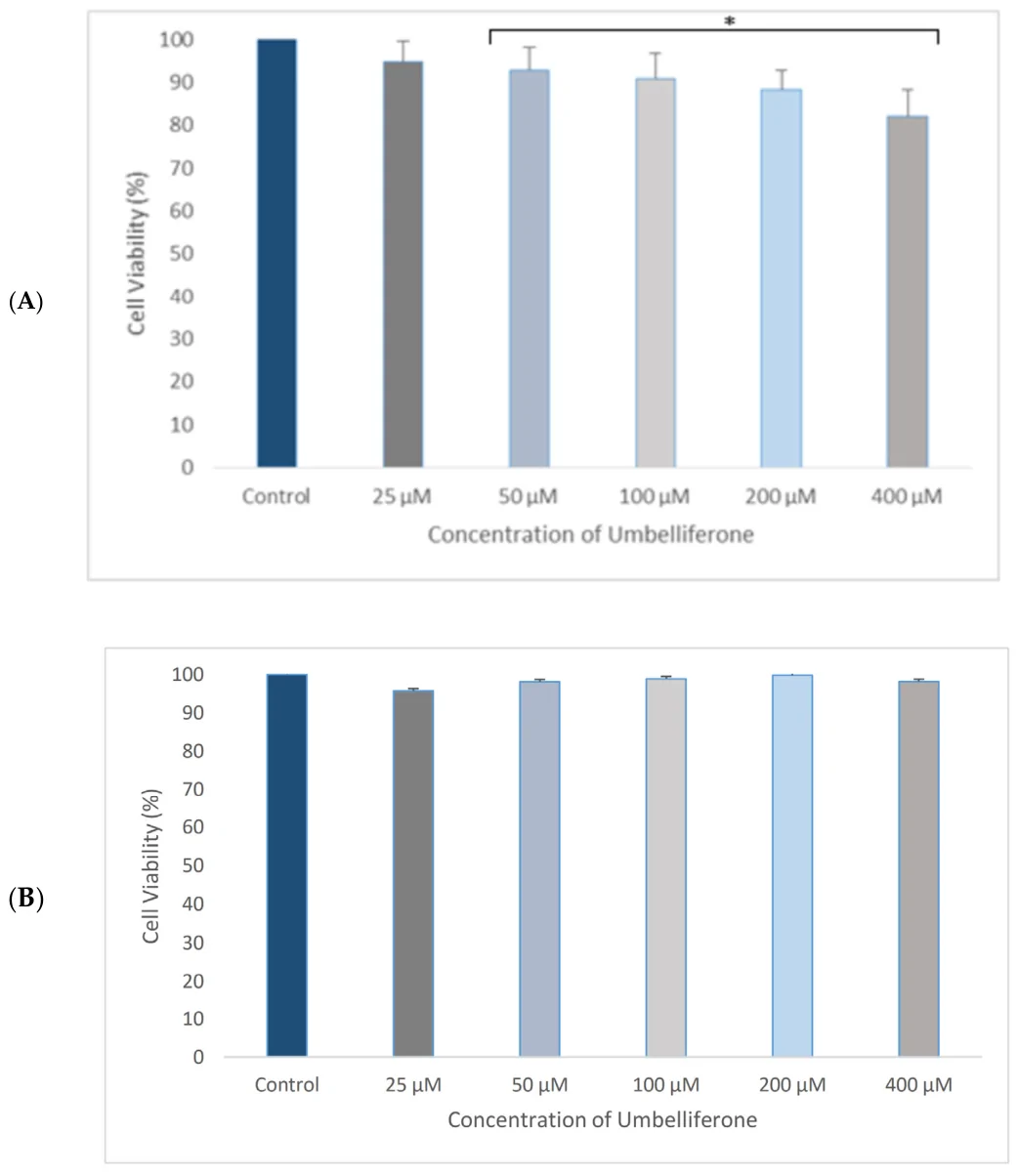
Effect of umbelliferone on cell viability in (**A**) MCF-7 breast cancer cells and (**B**) BJ normal fibroblasts following 24 h of treatment, as determined by the MTT assay. Data are presented as mean ± SD from three independent biological experiments (*n* = 3), each performed in technical triplicate. Statistical significance was evaluated using the Kruskal–Wallis test followed by Dunn’s multiple-comparison post hoc test. An * indicates a statistically significant difference from the untreated control (adjusted *p* < 0.05). For MCF-7 cells, Dunn’s adjusted *p* values versus the untreated control were 0.7970, 0.0437, 0.0121, <0.0001, and <0.0001 for 25, 50, 100, 200, and 400 µM umbelliferone, respectively.

**Figure 6:**
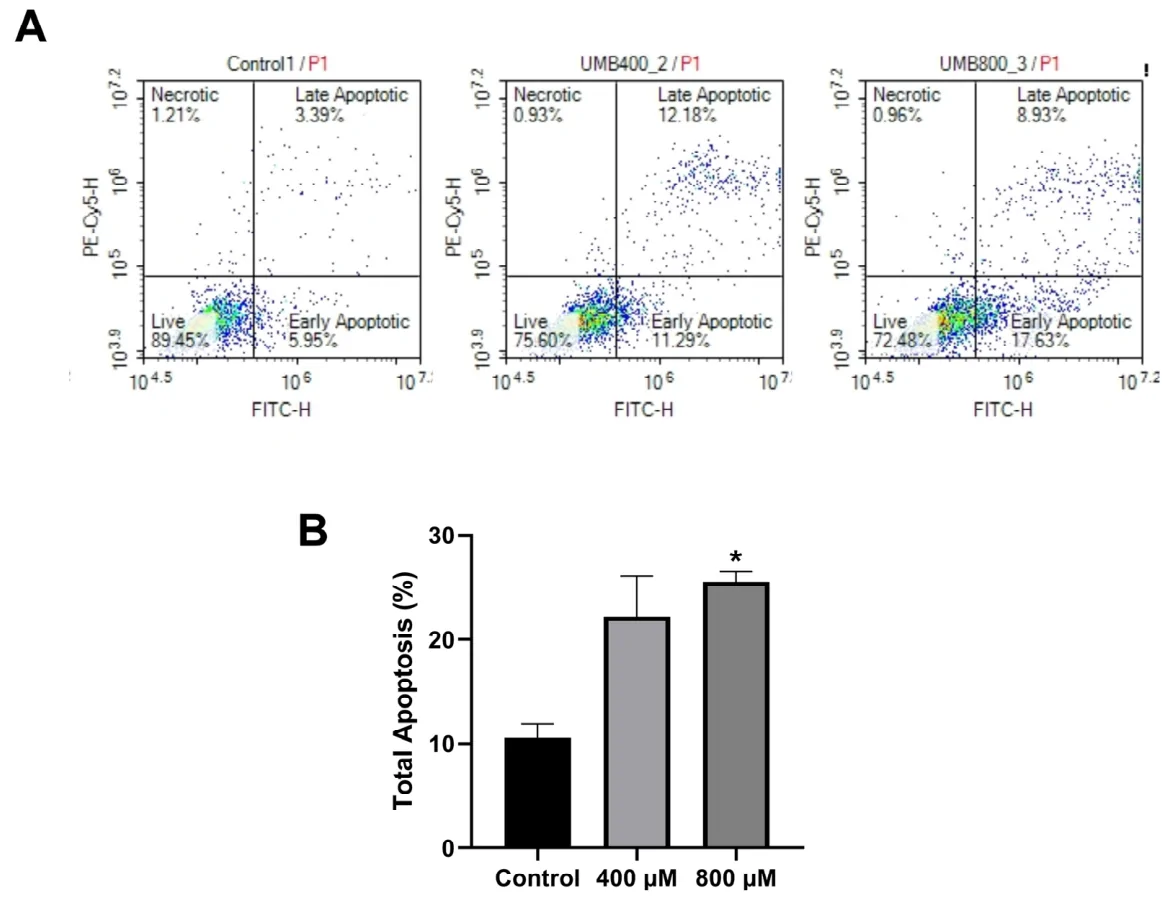
Effect of umbelliferone on apoptosis in MCF-7 breast cancer cells. (**A**) Representative flow cytometry dot plots of Annexin V-FITC/PI staining in untreated control cells and cells treated with 400 or 800 µM umbelliferone. The quadrants represent viable, early apoptotic, late apoptotic, and necrotic cell populations according to Annexin V-FITC and PI staining. (**B**) Quantitative analysis of total apoptotic cells, calculated as the sum of early and late apoptotic populations. Data are presented as mean ± SD from three independent biological experiments (*n* = 3), each performed in technical triplicate. Statistical significance was evaluated using the Kruskal–Wallis test followed by Dunn’s multiple-comparison post hoc test. Dunn’s adjusted *p* values versus the untreated control were 0.0731 and 0.0122 for 400 and 800 µM umbelliferone, respectively. An asterisk indicates a statistically significant difference from the untreated control (adjusted *p* < 0.05).

**Figure 7:**
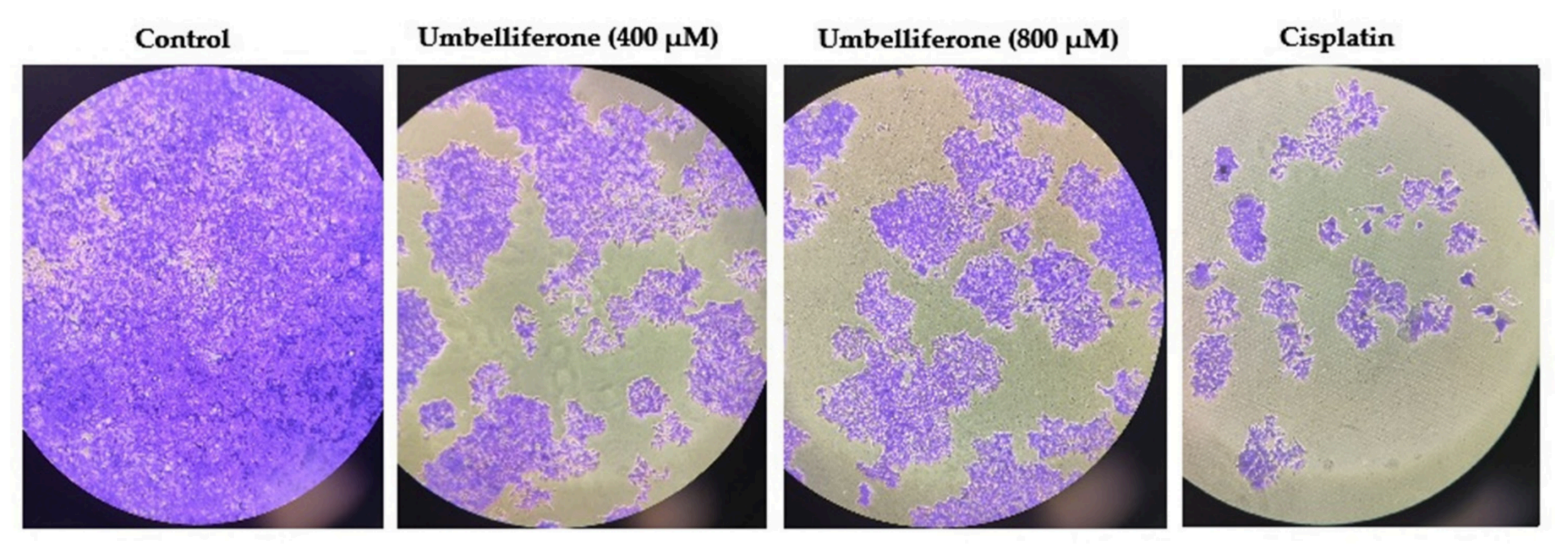
Representative microscopic images of colony formation in MCF-7 cells stained with crystal violet. Cells were treated with umbelliferone (400 and 800 µM) for 24 h, while cisplatin was used as a positive control and untreated cells served as the negative control. Images were acquired using an Olympus CK40 inverted microscope with a 10× objective.

**Figure 8:**
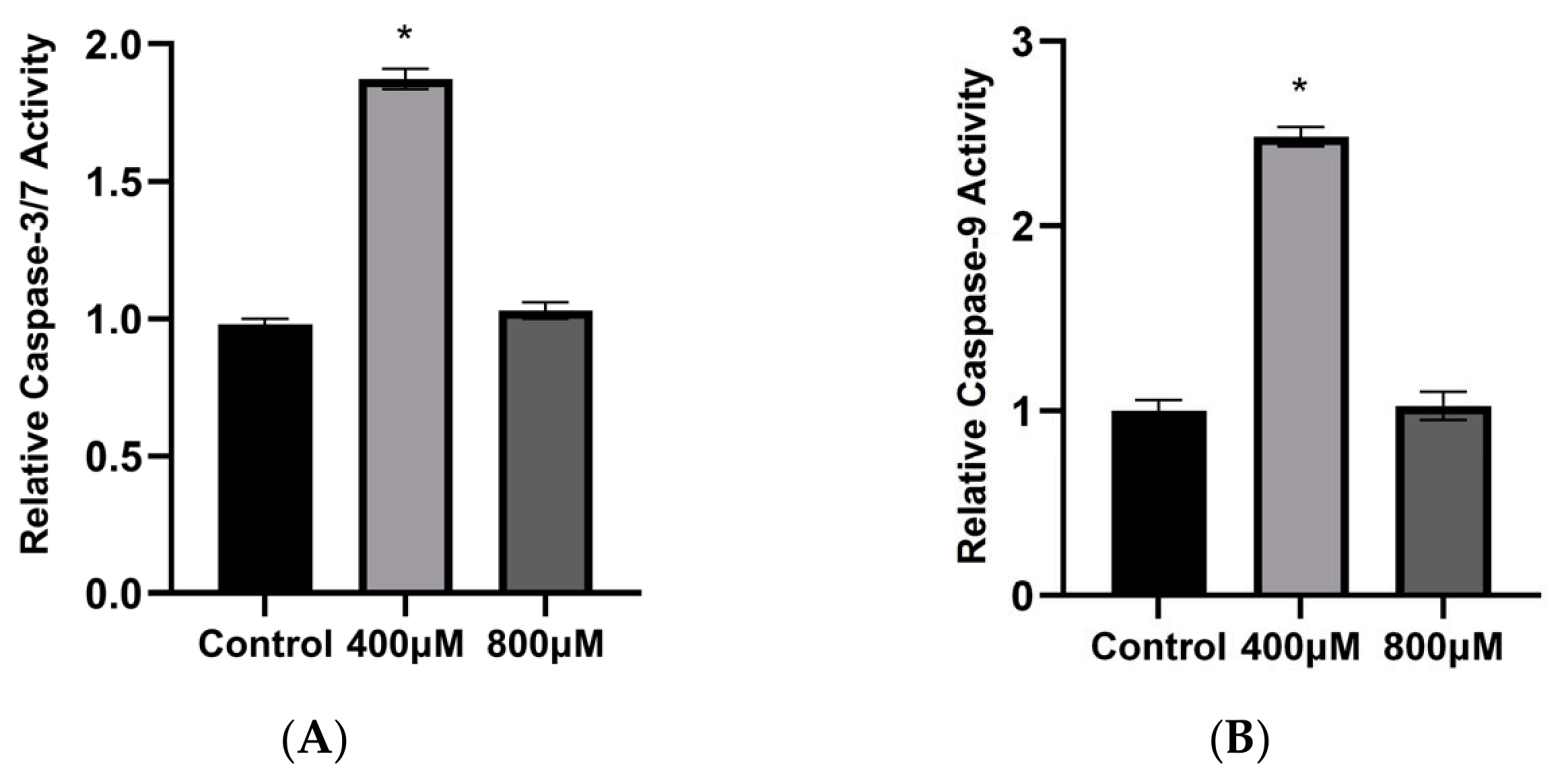
Effect of umbelliferone on caspase activity in MCF-7 cells. Relative (**A**) Caspase-3/7 and (**B**) Caspase-9 activities were determined following treatment with 400 and 800 µM umbelliferone. Data are presented as mean ± SD from three independent biological experiments (*n* = 3). * *p* < 0.05 compared with the untreated control. Given the Caspase-3-deficient nature of MCF-7 cells, the Caspase-3/7 assay signal was interpreted predominantly as Caspase-7-associated executioner caspase activity.

**Table 1:** Key binding site interactions of umbelliferone with AKT1, mTOR, PI3K*α*, and Caspase-7 identified from docking analysis.

Target Protein	Residue	Distance (Å)	Interaction Type
AKT1	SER205	1.661	Conventional Hydrogen Bond
	THR211	2.077	Conventional Hydrogen Bond
	ASN204	2.107	Conventional Hydrogen Bond
	LEU210	3.867	π–σ
	LEU210	3.994	π–σ
	TRP80	5.182	*π*–*π* Stacked
	TRP80	4.165	*π*–*π* Stacked
	TRP80	4.332	*π*–*π* Stacked
	LEU264	5.382	*π*–Alkyl
	LEU210	4.702	*π*–Alkyl
mTOR	ASN1898	2.048	Conventional Hydrogen Bond
	ASN1899	2.354	Conventional Hydrogen Bond
	LEU1900	2.172	Conventional Hydrogen Bond
	GLN1937	2.166	Conventional Hydrogen Bond
	LEU1900	3.875	π–σ
	LEU1900	3.600	π–σ
	LEU1900	3.354	π–σ
	LEU2204	5.253	*π*–Alkyl
PI3Kα	THR462	2.109	Conventional Hydrogen Bond
	ARG401	3.895	*π*–Cation
	ARG401	4.318	*π*–Cation
	THR462	3.796	π–σ
Caspase-7	THR463	3.016	Conventional Hydrogen Bond
	GLU516	4.289	*π*–Anion
	MET594	5.418	*π*–Sulfur
	PRO227	5.202	*π*–Alkyl

**Table 2:** Comparison of interacting residues between umbelliferone and co-crystallized ligands.

Target Protein	Overlapping Residues
AKT1	TRP80, LEU210
mTOR	LEU2204
Caspase-7	Not observed
PI3Kα	Not observed

**Table 3:** Hydrogen-bond interactions with occupancy >10% observed for the AKT1–umbelliferone and mTOR–umbelliferone complexes during molecular dynamics simulations.

Complex	Hydrogen Bond Interaction	Occupancy (%)
AKT1–umbelliferone	SER205 (main chain)–umbelliferone	49.10
mTOR–umbelliferone	No hydrogen bond with occupancy > 10%	Not applicable

**Table 4:** MM/PBSA energetic components of umbelliferone in complex with AKT1 and mTOR.

Protein	${∆E}_{electrostatic}$(kcal/mol)	van der Waals Force (kcal/mol)	${\Delta G}_{Poisson-Boltzmann}$(kcal/mol)	Surface Area (kcal/mol)	Gas Phase Energies (kcal/mol)	Solvation Free Energy (kcal/mol)	Polar Interactions (kcal/mol)	Nonpolar Interactions (kcal/mol)	Total MM/PBSA Estimate (kcal/mol)
AKT1	−0.02 ± 0.14	−0.01 ± 0.01	0.10 ± 8.05	−0.92 ± 0.0	−0.05 ± 0.14	−1.72 ± 8.06	0.20 ± 7.50	−0.92 ± 0.01	−3.73 ± 8.05
mTOR	−0.01 ± 0.89	−0.01 ± 0.02	−0.18 ± 6.92	−0.92 ± 0.01	−0.01 ± 0.88	−1.09 ± 6.91	−0.18 ± 6.89	−0.92 ± 0.01	−1.11 ± 6.90

**Table 5:** Colony formation following umbelliferone treatment in MCF-7 cells.

Groups	Number of Countable Colonies (mean ± SD)	Colony-Forming Efficiency (CFE, %)
Control	ND	ND
Cisplatin (40 µg/mL)	2.7 ± 1.2	5.3 ± 2.3
Umbelliferone (400 µM)	7.7 ± 0.6	15.3 ± 1.2
Umbelliferone (800 µM)	6.0 ± 0.0	12.0 ± 0.0

CFE, colony-forming efficiency; ND, not determined. Individual colonies in the untreated control group could not be reliably quantified because of extensive colony merging. Colonies containing ≥ 50 cells were considered countable colonies.

## Data Availability

The original contributions presented in this study are included in the article/Supplementary Materials. Further inquiries can be directed to the corresponding author.

## Supplementary Materials

The following supporting information can be downloaded at https://www.mdpi.com/article/10.3390/ph19101536/s1 Table S1. Time-window analysis of MM/PBSA energetic estimates for the AKT1–umbelliferone and mTOR–umbelliferone complexes. Values are presented as mean ± SD (kcal/mol) for five consecutive 10 ns intervals between 50 and 100 ns.

## Abbreviations

The following abbreviations are used in this manuscript:

HR^+^	Hormone receptor-positive
TNBC	Triple negative breast cancer
PI3Kα	phosphoinositide 3-kinase alpha
MTT	3-(4,5-Dimethylthiazol-2-yl)-2,5-Diphenyltetrazolium Bromide
ADME	Absorption, distribution, metabolism and elimination
MD	Molecular dynamics
RMSD	Root mean square deviation
RMSF	Root mean square fluctuation
R_g_	Radius of gyration
MM/PBSA	Molecular Mechanics/Poisson–Boltzmann Surface Area
TPSA	Topological polar surface area
IC_50_	Half-maximal inhibitory concentration
CaFE	Calculation of Free Energy
APBS	Adaptive Poisson–Boltzmann Solver
