# Supplementary material for: Biological Effects of Umbelliferone in MCF-7 Breast Cancer Cells: Integrated In Vitro and Computational Analyses
Source: Pharmaceuticals (Basel). 2026 Sep 28;19(10):1536. doi: 10.3390/ph19101536 (PMC13708397; doi:10.3390/ph19101536)
Supplement: Supplementary file 1 [file pharmaceuticals-19-01536-s001.zip › pharmaceuticals-4586618-supplementary.pdf]

Supporting Information

Biological Effects of Umbelliferone in MCF-7 Breast Cancer Cells: Integrated In Vitro and Computational Analyses

Table S1. Time-window analysis of MM/PBSA energetic estimates for the AKT1–umbelliferone and mTOR–umbelliferone complexes. Values are presented as mean ± SD (kcal/mol) for five consecutive 10 ns intervals between 50 and 100 ns.

Time window (ns)	AKT1–umbelliferone Total MM/PBSA Estimate (kcal/mol)	mTOR–umbelliferone Total MM/PBSA Estimate (kcal/mol)
50–60	−0.797 ± 6.821	−0.981 ± 7.913
60–70	−2.095 ± 6.796	−1.004 ± 5.821
70–80	−2.787 ± 8.114	−1.162 ± 6.113
80–90	−3.551 ± 8.059	−1.103 ± 6.931
90–100	−3.377 ± 7.305	−1.115 ± 5.487
